# Critical Effect of H_2_O_2_ in the Agar Plate on the Growth of Laboratory and Environmental Strains

**DOI:** 10.1128/spectrum.03336-22

**Published:** 2022-11-02

**Authors:** Motoyuki Watanabe, Kensuke Igarashi, Souichiro Kato, Yoichi Kamagata, Wataru Kitagawa

**Affiliations:** a Graduate School of Agriculture, Hokkaido University, Sapporo, Hokkaido, Japan; b Graduate School of Global Food Resources, Hokkaido University, Sapporo, Hokkaido, Japan; c Bioproduction Research Institute, National Institute of Advanced Industrial Science and Technology (AIST), Sapporo, Hokkaido, Japan; d Bioproduction Research Institute, National Institute of Advanced Industrial Science and Technology (AIST), Tsukuba, Ibaraki, Japan; Connecticut Agricultural Experiment Station

**Keywords:** hydrogen peroxide, agar plate, growth, colony formation, laboratory strains, environmental strains

## Abstract

We previously showed that autoclaving in preparing agar media is one of the sources of hydrogen peroxide (H_2_O_2_) in the medium. This medium-embedded H_2_O_2_ was shown to lower the total colony count of environmental microorganisms. However, the critical concentrations of H_2_O_2_ detrimental to colony formation on the agar plate remain largely undetermined. Herein, we elucidated the specific effect of H_2_O_2_ on microbial colony formation on solid agar medium by external supplementation of varying amounts of H_2_O_2_. While common laboratory strains (often called domesticated microbes) formed colonies in the presence of high H_2_O_2_ concentrations (48.8 μM or higher), microbes from a freshwater sample demonstrated greatly decreased colony counts in the presence of 8.3 μM H_2_O_2_. This implies that environmental microbes are susceptible to much lower concentrations of H_2_O_2_ than laboratory strains. Among the emergent colonies on agar plates supplemented with different H_2_O_2_ concentrations, the relative abundance of betaproteobacterial colonies was found to be lower on plates containing higher amounts of H_2_O_2_. Further, the growth of the representative betaproteobacterial isolates was completely inhibited in the presence of 7.2 μM H_2_O_2_. Therefore, our study clearly demonstrates that low micromolar levels of H_2_O_2_ in agar plates critically affect growth of environmental microbes, and large portions of those are far more susceptible to the same than laboratory strains.

**IMPORTANCE** It is well-known that most of environmental microorganisms do not form colonies on agar medium despite that agar medium is the commonly used solidified medium. We previously demonstrated the negative effects of H_2_O_2_ generation during agar medium preparation on colony formation. In the present study, we investigated the independent effect of H_2_O_2_ on microbial growth by adding different concentrations of H_2_O_2_ to agar medium. Our results demonstrate for the first time that even low micromolar levels of H_2_O_2_ in agar plates, that are far lower than previously recognized as significant, adversely affect colony number obtained from freshwater inoculum.

## INTRODUCTION

Hydrogen peroxide (H_2_O_2_), a reactive oxygen species, is destructive to microorganisms (1, 2). Microbial susceptibility have long been investigated in a variety of laboratory cultures (3–14). These studies demonstrate the effect exerted by several hundred micromolar to millimolar levels of supplementary H_2_O_2_ on bacterial survival. For instance, the Escherichia coli K-12 strain W3110 survives after the 15-min treatment with 10 mM H_2_O_2_ (5), and the survival rate of E. coli K-12 strain AB1157 was below 5% after the 15-min treatment with 25 mM H_2_O_2_ (14). The majority of these studies exposed bacterial cells to H_2_O_2_ in liquid medium prior to spreading them on solid medium. This resulted in H_2_O_2_ stress that was exerted on bacterial cells in liquid medium, being evaluated on solid medium. Because the H_2_O_2_ content of the solid agar media was not determined, the H_2_O_2_ stress for microbial cells on solid (more specifically, agar) media are poorly assessed.

Agar has been commonly used as a growth media solidifier since the 1890s and modern microbiology would not exist without its extensive contributions to the field. Nonetheless, that agar medium does not support the growth of most environmental microbes is widely recognized, and this phenomenon is often referred to as the “great plate count anomaly” (15). Several factors that contribute to this effect in agar media have been established thus far, including the role played by H_2_O_2_ generation during agar media preparation as previously reported by us (16, 17).

We demonstrated that autoclaving agar and phosphate in the same container during the media preparation (PT protocol, phosphate-together) resulted in the generation of H_2_O_2_ that remained entrenched within the medium (16). This retention of H_2_O_2_ in media is believed to be one of the factors responsible for the lowered colony counts of environmental microbes (15–18). H_2_O_2_ generation increases in a phosphate concentration-dependent manner, and is accelerated by high pH and ammonium concentrations (17). The generation of H_2_O_2_ can thus be reduced by autoclaving agar and phosphate separately (PS protocol, phosphate-separate) (16, 17). Comparative analyses of these two recipes using bacterial inoculums derived from several environmental samples revealed that the colony yield of PS plates was at least twice as high as that of PT plates (15–18). This was accompanied by a higher ratio of phylogenetically novel isolates on PS plates than that observed on PT plates (16, 18, 19). These results imply that the use of PT medium critically affects microbial colony formation, and the cultivability of hitherto-uncultivated microorganisms. The above-mentioned studies utilized PYG agar medium (containing peptone, yeast extract, and glucose as primary carbon and energy sources) as a model medium that potentially contains H_2_O_2_ at concentrations of ~15 μM or higher when plates are prepared using the PT protocol (16, 17).

While we previously reported that PT plates yield fewer colonies than that observed on PS plates, the effect might not be a consequence solely attributable to higher H_2_O_2_ content. This is because PT plates are discussed to contain other growth inhibiting substances that are generated during the autoclaving process (17). Thus, a simple comparison of growth between PT and PS plates does not accurately reflect the independent effects of H_2_O_2_.

The present study investigates the independent effect of H_2_O_2_ contained within agar medium on the growth of laboratory and environmental microbes. The modulation of H_2_O_2_ levels in media allowed investigation of the specific effects of H_2_O_2_ on microbial growth, independent of the effects exerted by other growth inhibiting substances that may have been generated during agar media preparation (17).

## RESULTS

### Evaluation of various microbial sensitivities to H_2_O_2_ in agar medium.

PYG plates without supplemental phosphate (PW protocol, without phosphate) that have been reported to yield less H_2_O_2_ than those prepared by the PS protocol (generated H_2_O_2_: PT≫PS>PW) were prepared (16). The concentration dependent effects of H_2_O_2_ on colony formation were assessed using PW plates supplemented with varying amounts of H_2_O_2_ before solidification.

The sensitivities of laboratory strains (see Materials and Methods) to H_2_O_2_ in agar plates was tested by spreading cell suspensions on PW plates supplemented with H_2_O_2_ (Table 1). The findings revealed that while *Sphingomonas* and Pseudomonas strains managed to grow on plates supplemented with 48.8 μM H_2_O_2_, Escherichia and *Rhodococcus* strains showed growth at a much higher concentration of 85.3 μM. Further, *Bacillus* strain demonstrated growth even at 225 μM H_2_O_2_, the highest concentration tested. All of these commonly studied species were thus capable of growing on agar plates containing at least 48.8 μM H_2_O_2_, which is approximately twice the H_2_O_2_ concentration detected in conventional PT plates (16, 17).

**TABLE 1:** The growth of various bacterial strains on the PW plates with different H_2_O_2_ concentrations^*a*^

	Strain	H_2_O_2_ concn of the PW plate (μM)						
Bacterial type		0.6	2.9	7.2	13.3	48.8	85.3	225.0
Common laboratory species	Escherichia coli K-12	+	+	+	NT	+	+	−
	Pseudomonas putida JCM 6157	+	+	+	NT	+	−	−
	Sphingomonas paucimobilis NBRC 13935^T^	+	+	+	NT	+	−	−
	Rhodococcus erythropolis JCM 3201^T^	+	+	+	NT	+	+	−
	Bacillus subtilis Subsp. Subtilis Str. 168	+	+	+	NT	+	+	+
Isolated in this study	OS-1	+	+	−	−	−	−	−
	OS-4	+	+	−	−	−	−	−
Isolated in the previous study	SO-S41	+	+	+	NT	−	−	−

a +, growth; −, no growth; NT = not tested.

### Comparison of colony diversity and frequency obtained from environmental sample at varying H_2_O_2_ concentrations.

**FIG 1:**
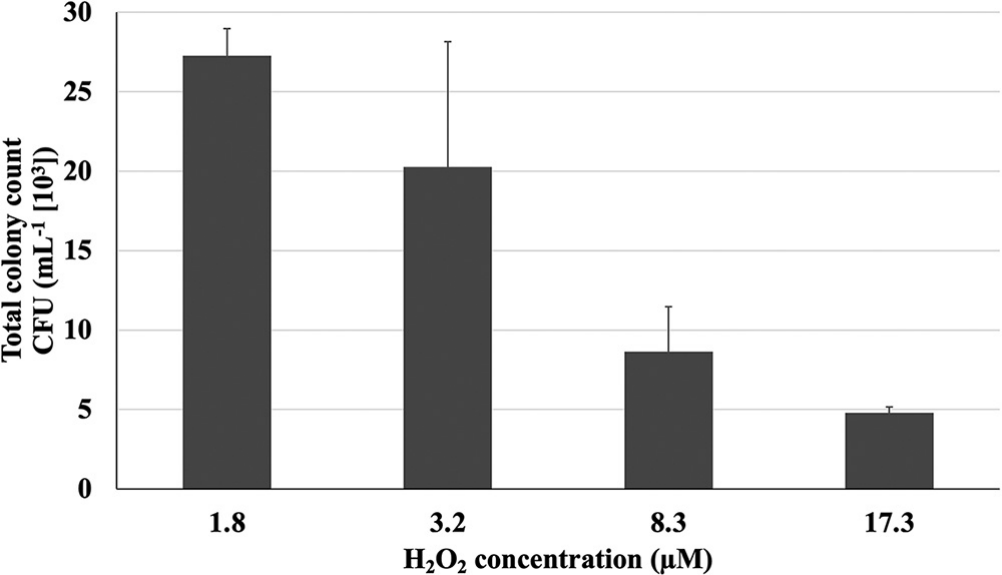
Total colony count of emergent colonies from the freshwater sample on PW plates with four different H_2_O_2_ concentrations. The number of colonies were counted after 10 days of incubation at 20°C in dark. CFU counts are averages from four replicate agar plates and error bars represent standard deviations.

The freshwater microbial sample was inoculated on PW plates with four different H_2_O_2_ concentrations to elucidate its effect on microbial growth in the context of colony frequency and diversity. Supplemental H_2_O_2_ was added postautoclaving to ensure that plates only differed with respect to their final H_2_O_2_ concentrations. The highest H_2_O_2_ concentration of 17.3 μM was comparable to the H_2_O_2_ concentration present in PT plates, as previously reported (16). The cultivation was performed in quadruplicate and the CFU was calculated with the standard deviation. The CFU were found to decrease with increasing H_2_O_2_ concentrations in PW plates (Fig. 1). This was evident from the finding that 20% of the CFU that grew on the 1.8 μM H_2_O_2_ plates were lost on the 3.2 μM plates. Further, more than 60% were lost on the 8.3 μM plates, and more than 80% was lost on the 17.3 μM plates.

**FIG 2:**
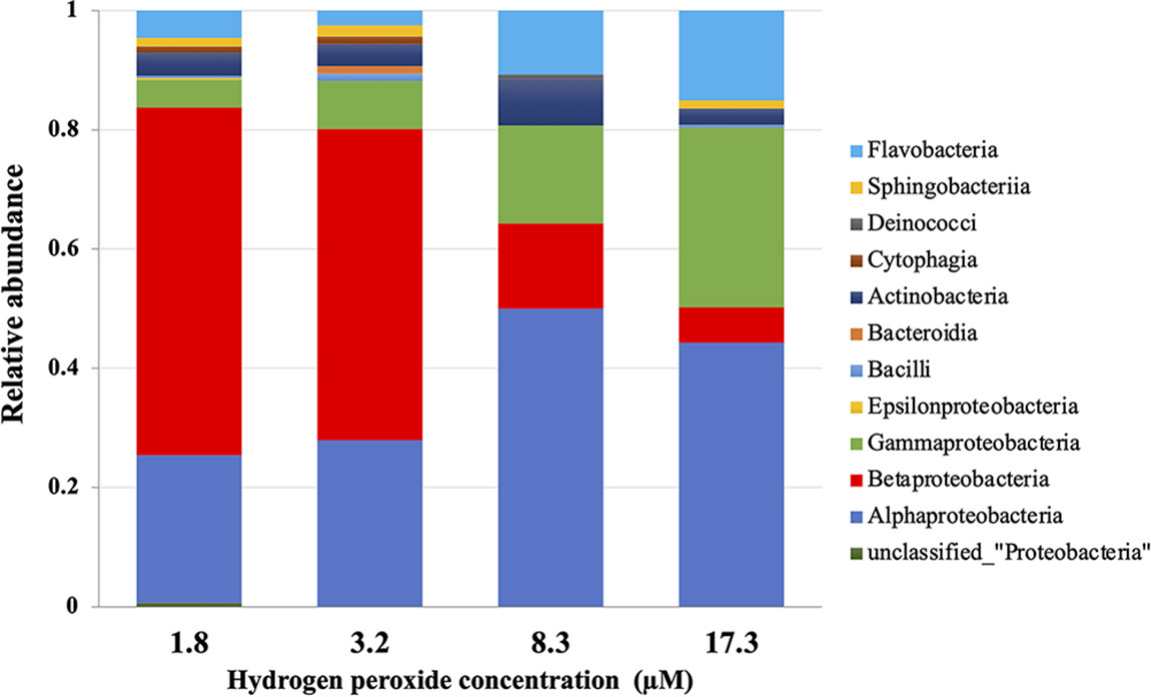
Comparison of the class level relative abundance of emergent colonies on PW plates with four different H_2_O_2_ concentrations. Colonies were randomly selected from the plates and identified by analyzing the amplified partial sequence of 16S rRNA gene using RDP classifier.

The H_2_O_2_ concentration also affected the taxonomic composition of the colonies, as depicted in Fig. 2 in terms of microbial diversity at the class level. While, the relative abundance of the classes *Gammaproteobacteria*, *Flavobacteria*, and *Alphaproteobacteria* was much higher on plates that contained greater amounts of H_2_O_2_, a decrease in the relative abundance of the class *Betaproteobacteria* was evident when H_2_O_2_ concentrations were comparatively higher.

### Isolation and characterization of microbes that are highly sensitive to H_2_O_2_.

Based on the above-mentioned results, we attempted to obtain microbes that were highly sensitive to low micromolar levels of H_2_O_2._ More specifically, betaproteobacterial colonies were selectively collected among the identified colonies which depicted Fig. 2.

Of the various betaproteobacterial strains isolated from the 1.8-μM H_2_O_2_ plates, OS-1 and OS-4 were selected for further experimentation.

Analysis of the 16S rRNA gene sequence using the NCBI database (https://blast.ncbi.nlm.nih.gov/Blast.cgi) revealed that the strain OS-1 shared approximately 99% identity with certain *Rhodoferax* strains, and that the strain OS-4 was approximately 99% identical to certain *Curvibacter* strains. This implies that they may belong to these genera, both of which are classified under the family *Comamonadaceae*. Notably, the family *Comamonadaceae* also demonstrated the most obvious decrease in CFU with increasing H_2_O_2_ concentrations (Fig. S1; Table S1).

**FIG 3:**
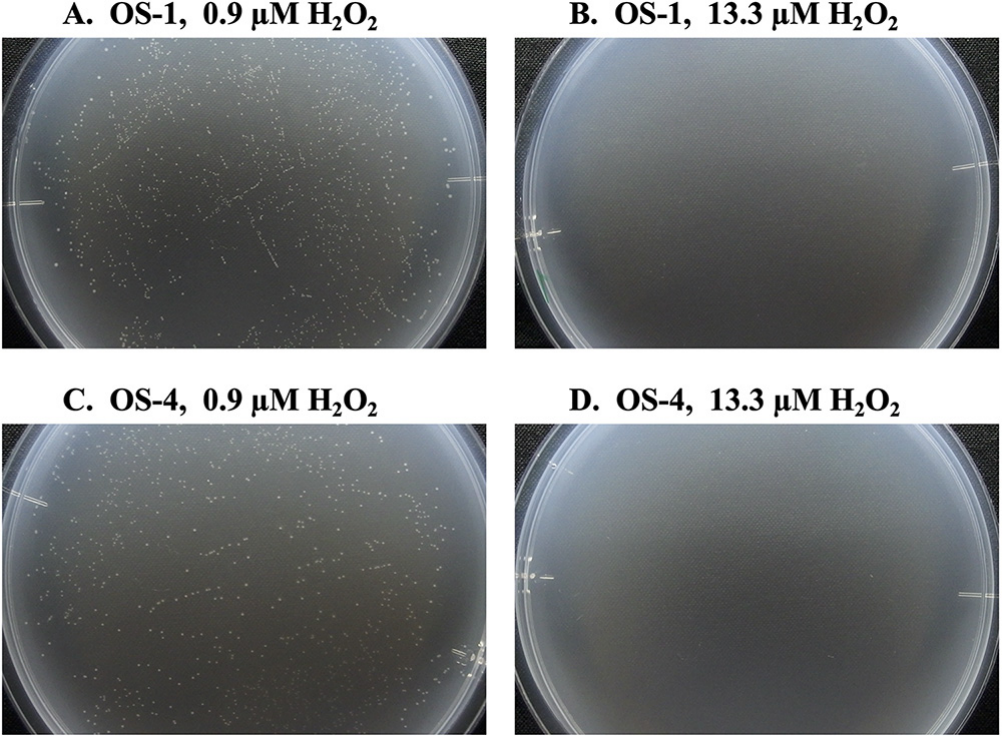
Colony formation of OS-1 and OS-4 on PW plates with 0.9 μM (A and C) and 13.3 μM (B and D) of H_2_O_2_. Pictures were taken after 5 days of incubation at 20°C. Cultivation experiment was performed in pentaplicates and the representative is shown.

The reproducibility of the results on H_2_O_2_ sensitivity of these two strains was confirmed after isolation and long-term preservation. Both strains were found to form colonies on PW plates supplemented with 0.9 μM H_2_O_2_; however, colony formation was completely abrogated on plates containing 13.3 μM H_2_O_2_, a concentration which is comparable to that present in conventional PT plates (Fig. 3).

Further, both OS-1 and OS-4 failed to form single colonies on plates supplemented with 7.2 μM H_2_O_2_ (Table 1). This indicates that their threshold of H_2_O_2_ sensitivity is substantially lower than the amount of H_2_O_2_ in conventional PT plates.

Additionally, the alphaproteobacterial strain SO-S41, which grows on PS plates but not on PT plates, was also tested for H_2_O_2_ sensitivity (18) (Table 1). The strain formed single colonies on the plates supplemented with 7.2 μM H_2_O_2_, which is indicative of its lower sensitivity to H_2_O_2_ than that of OS-1 and OS-4.

## DISCUSSION

The present study aimed to shed light on the specific effects of H_2_O_2_ on the cultivability (colony formation) of environmental isolates and laboratory strains. Because our previous studies that utilized PT and PS plates were limited by its inability to exclude the effects of other interfering substances that may have been detrimental to colony forming capacity, we used growth media that was prepared using an identical protocol to ensure that the PW plates only differed with respect to their H_2_O_2_ contents. These were prepared by supplementing agar medium postautoclaving with H_2_O_2_, thus allowing the manipulation of the H_2_O_2_ content, and were subsequently used to assess the H_2_O_2_ susceptibilities of various microbes (Table 1). All the tested laboratory strains successfully formed single colonies on PW plates containing 48.8 μM H_2_O_2_, thus indicating that their tolerance to H_2_O_2_ in agar plates was higher than the amount of H_2_O_2_ in conventional PT plates. The high tolerance of these strains may contribute to their growth on artificial media that contain a certain amount of H_2_O_2_, which possibly further led to their establishment as common laboratory strains.

The majority of studies that investigated microbial susceptibility to H_2_O_2_ exposed bacterial cells to millimolar levels of H_2_O_2_ in liquid medium prior to spreading on solid medium containing unknown amounts of H_2_O_2_ (3–14). In contrast, we analyzed the direct effect of H_2_O_2_ in agar plates on inoculated cells, so as to be able to determine the vulnerability of bacterial cells to the representative concentrations of reactive oxygen species, when plated on solidified media. The results revealed that the well-known laboratory strains of Escherichia, Pseudomonas, *Rhodococcus*, and *Sphingomonas* were able to form colonies at concentrations up to 48.8 to 85.3 μM H_2_O_2_ in agar plates, but were not able to form colonies in the presence of 225 μM H_2_O_2_ (Table 1), which is in sharp contrast to previous findings that report resistance even to millimolar amounts of H_2_O_2_ in liquid medium. This significant difference between previous reports and our study probably exists on account of the previous studies have reported survivability in the presence of H_2_O_2_ in liquid medium, while our study demonstrated the effect of H_2_O_2_ on colony formation. Ours, therefore, is the first study that demonstrates microbial susceptibility of pure strains, including E. coli strains, to potentially lethal concentrations of H_2_O_2_ on agar media.

We further hypothesized that the colony forming ability of many environmental microbes would potentially be significantly affected by H_2_O_2_ in agar plates. Given that 15 μM or higher H_2_O_2_ in agar plates, a concentration previously detected in PT media by us, had an inhibitory effect on the growth of environmental microbes (16, 17), the effect of lower H_2_O_2_ concentrations was investigated in the present study. PW plates with four different H_2_O_2_ concentrations, namely, 1.8 μM, 3.2 μM, 8.3 μM, and 17.3 μM, were prepared and inoculated with a freshwater sample that served as a bacterial source. The number of emergent colonies were subsequently counted, which clearly revealed that H_2_O_2_ concentrations in agar plates were inversely related to colony numbers (Fig. 1). As shown in Fig. 1, a remarkable decrease in CFU was observed on the 8.3-μM plate, an H_2_O_2_ concentration which was obviously lower than the concentration previously detected in PT plates (~15 μM) (15, 16). These results demonstrate the levels of H_2_O_2_ to which environmental microbes are sensitive for the first time.

The class level abundance of the colonies was directly influenced by H_2_O_2_ content. The identification of colonies on different plates revealed that colony variety differed between different H_2_O_2_ concentrations. The class *Betaproteobacteria* demonstrated a continuous decline as H_2_O_2_ concentrations increased (Fig. 2), which is indicative of the existence of H_2_O_2_ sensitive strains among *Betaproteobacteria*. This assisted us substantially in the isolation of the betaproteobacterial strains OS-1 and OS-4 from plates supplemented with 1.8 μM H_2_O_2_. Both these strains failed to grow in the presence of 7.2 μM H_2_O_2_, a concentration lower than that present in PT plates (Table 1), even after repetitive subculture in the laboratory, implying that they lack the ability to colonize conventional PT plates. Notably, in sharp contrast to the laboratory strains, both OS-1 and OS-4 were observed to have higher susceptibilities to H_2_O_2_ in agar plates.

Preliminary genome-sequencing analysis revealed the presence of the putative catalase gene in both the OS-1 and OS-4 genomes; however, both strains demonstrated negative catalase activity (data not shown). Similarly, the previously isolated alphaproteobacterial strain SO-S41 that grows on PS plates, but not on PT plates (18), was also found to contain a putative catalase gene in its genome (20). Taken together, our results indicate that microbes that possess catalase genes in their genomes could nonetheless be sensitive to low micromolar levels of H_2_O_2_ in agar plates.

In order to further evaluate the effect of plate-embedded H_2_O_2_ on the growth of environmental microbes, more samples from different locations and time period should be collected and examined. However, we successfully discovered aerobic microbes which are sensitive to low micromolar of H_2_O_2_ from our freshwater sample. Moreover, environmental microbes that grow on PS plates but not on PT plates have been sourced from water, sludge, sediment, and soil (16–19), the majority (though not all) of which are in all likelihood sensitive to low micromolar levels of H_2_O_2_ in agar plates.

In the present study, we prepared agar media using 1.8 μM H_2_O_2_ as the lowest concentration, which yielded the highest CFU from freshwater sample inoculums. However, given that the H_2_O_2_ content of freshwater samples has been reported to be in the nanomolar order (21) and in view of our evidence on the sensitivity of environmental microbes to H_2_O_2_, one may reasonably expect the existence of a number of microbes in these samples that are incapable of growing even on plates supplemented with 1.8 μM H_2_O_2_. The analysis of microbes with far more sensitivity to H_2_O_2_ therefore requires the preparation of plates with nanomolar levels of H_2_O_2_. While this may be achieved by the addition of catalase or pyruvate that reduce H_2_O_2_ to extremely low levels (17, 22–25), the technique cannot be applied to control H_2_O_2_ concentrations. The development of novel techniques that can successfully regulate H_2_O_2_ content in the nanomolar order are therefore essential for the analysis of more sensitive microbes.

Our results demonstrated that the low micromolar levels of H_2_O_2_ in agar plates critically affected the growth of environmental microbes, which further implied that the micromolar levels of H_2_O_2_ generated during media preparation may be one of the causes of the “great plate count anomaly” (15).

Further studies on H_2_O_2_ sensitive strains, including a detailed analysis of the extent of their sensitivities to different H_2_O_2_ levels, as well as of the activities of catalase and other hydrogen peroxide-degrading enzymes encoded by their genomes, might aid elucidation of the mechanisms involved in H_2_O_2_ susceptibility. This may prove to be useful in the development of technological advancements that will permit the isolation of novel environmental microbes in the future.

## MATERIALS AND METHODS

### Environmental sample source and collection.

Environmental samples were collected from the current beside Ono pond, a pond located at Hokkaido University, Sapporo, Japan (43°07’N, 141°34’E) in order to isolate microbes sensitive to low micromolar levels of H_2_O_2_. The sediment surface was disturbed using an autoclave-sterilized ladle, and the water above containing floating sediment particles was subsequently collected into an autoclave-sterilized plastic laboratory bottle. The collected sample was immediately placed on ice and stored at 4°C until further use in cultivation experiments.

### Preparation of culture medium.

PYG medium (containing peptone, yeast extract, and glucose) was prepared using the PW protocol for microbial cultivation from the environmental sample, as previously described by Tanaka et al. (16). Two solutions (solutions A and B) were prepared and autoclaved separately in different containers and subsequently mixed prior to solidification of the agar medium in a Petri dish. The pH of solution A containing 2.27 mM (NH_4_)_2_SO_4_, 0.2 mM MgSO_4_, and 45 μM CaCl_2_, was adjusted to 8.1 before the addition of 16 g L^−1^ of Bacto agar. Solution B contained 0.1 g L^−1^ of Bacto peptone, Bacto yeast extract, and glucose (pH 6.7). After mixing solutions A and B, H_2_O_2_ was added as required. Briefly, after cooling the autoclaved AB mixture to approximately 50°C, commercially available H_2_O_2_ was added to the mixture at incremental concentrations ranging from 5 to 400 μM, before immediate solidification in a Petri dish. The final concentration of retained H_2_O_2_ in the agar plates was detected to be approximately 40% to 60% of that which was originally supplemented.

### Cultivation and identification of microbes from environmental samples.

Prior to initiation of the cultivation experiments, the environmental sample was inverse-mixed in a plastic bottle and left for 1 h to allow large particles to settle. The liquid at the top was separated and serially diluted in a 10-fold series from 10^−1^ to 10^−4^ in sterile distilled water. From each dilution, 50 μL was spread on PW plates containing either 1.8 μM, 3.2 μM, 8.3 μM, or 17.3 μM H_2_O_2_. Each dilution was inoculated in quadruplicate for each H_2_O_2_ concentration, and the plates were subsequently incubated at 20°C in dark for 10 days prior to estimation of CFU.

At least 100 colonies from each H_2_O_2_ concentration were randomly chosen for identification (1.8 μM:384 colonies, 3.2 μM:288 colonies, 8.3 μM:192 colonies, 17.3 μM:384 colonies). Briefly, the partial region of 16S rRNA genes was PCR amplified using KOD FX Neo DNA polymerase (TOYOBO) and the primers set 10F’ (5′-AGAGTTTGATCMTGGCTCAG-3′) and 1492R (5′-TACGGYTACCTTGTTACGACTT-3′). The PCR products were prepared for sequencing using the BigDye Terminator cycle V3.1 sequencing kit (Thermo Fisher Scientific), and subsequently sequenced using the Applied Biosystems 3500XL genetic analyzer (Thermo Fisher Scientific) with the primer 341F (5′-CCTACGGGAGGCAGCAG-3′´). Each colony was identified using the Ribosomal Database Project (RDB) classifier (26) with a confidence threshold of 80%. The full-length sequence of the amplified 16S rRNA gene obtained from the isolates OS-1 and OS-4 was determined and used for phylogenetic analysis.

### Detection of H_2_O_2_ concentration in the medium.

H_2_O_2_ concentration in the agar medium was estimated by freezing the medium overnight at −80°C, followed by thawing in the dark for 3 h at room temperature. The liquid inside the agar medium was extracted onto the surface of the medium by syneresis, and was subsequently collected and diluted as liquid samples prior to detection of the H_2_O_2_ concentration.

The H_2_O_2_ concentration was analyzed by combining different aspects of the protocols previously published by Jiang et al. and Tanaka et al. (16, 27). In brief, freshly prepared 2× H_2_O_2_ assay reagent (200 mM sorbitol, 200 μM xylenol orange, 500 μM Fe(NH_4_)_2_(SO_4_)_2_·6H_2_O, and 50 mM H_2_SO_4_) was added to the same volume of the liquid sample which was collected from the thawed frozen plate. Prior to this, an H_2_O_2_-eliminated blank for each liquid sample was prepared by adding bovine liver catalase to the liquid samples in order to eliminate H_2_O_2_, followed by incubation at room temperature for 40 min. The absorbance was subsequently read at 560 nm and compared with that of the H_2_O_2_ standard solution, the concentration of which was determined using the extinction coefficient of 43.6 M^−1^·cm^−1^ at 240 nm. For each analysis, an average of triplicate measurements were made.

### Evaluation of microbial sensitivity to H_2_O_2_.

Eight bacterial strains were cultured on agar plates containing six different H_2_O_2_ concentrations. Five frequently utilized bacterial species included E. coli K-12, Pseudomonas putida JCM 6157, Sphingomonas paucimobilis NBRC 13935^T^, Rhodococcus erythropolis JCM 3201^T^, and Bacillus subtilis subsp. subtilis str. 168. The present study resulted in the isolation of two strains that were highly sensitive to H_2_O_2_, namely, OS-1 and OS-4. SO-S41 was an alphaproteobacterial strain that was isolated in our previous study (18). Each strain was cultured in PW liquid medium and harvested during the log phase. Cell suspensions were subsequently diluted, and 50 μL of the diluted suspensions were spread on PW plates containing either 0.6 μM, 2.9 μM, 7.2 μM, 13.3 μM, 48.8 μM, 85.3 μM, or 225 μM H_2_O_2_. Plates that were inoculated with E. coli were incubated at 37°C; those with Pseudomonas, *Sphingomonas*, *Rhodococcus*, and *Bacillus* strains at 28°C; those with OS-1 and OS-4 at 20°C; and those with SO-S41 at 25°C.

### Data availability.

The 16S rRNA gene sequences of strain OS-1 and OS-4 has been deposited in the GenBank/EMBL/DDBJ databases under accession numbers LC710547 and LC710548. The partial 16S rRNA gene sequences behind Fig. 2 are deposited under accession number LC734098-LC734900.
