# Supplementary material for: Critical Effect of H2O2 in the Agar Plate on the Growth of Laboratory and Environmental Strains
Source: Microbiol Spectr. 2022 Nov 2;10(6):e03336-22. doi: 10.1128/spectrum.03336-22 (PMC9769597; doi:10.1128/spectrum.03336-22)
Supplement: Supplemental file 1 — Supplemental material. Download spectrum.03336-22-s0001.pdf, PDF file, 0.2 MB [file spectrum.03336-22-s0001.pdf]

Supplemental Material

Critical effect of H_2O_2 in the agar plate on the growth of laboratory and environmental strains

Motoyuki Watanabe, Kensuke Igarashi, Souichiro Kato, Yoichi Kamagata and Wataru Kitagawa

Content

FIG S1 Phylogenetic distribution of the identified colonies classified at the family level.

TABLE S1 The count of identified colonies which was used to plot Fig. S1.

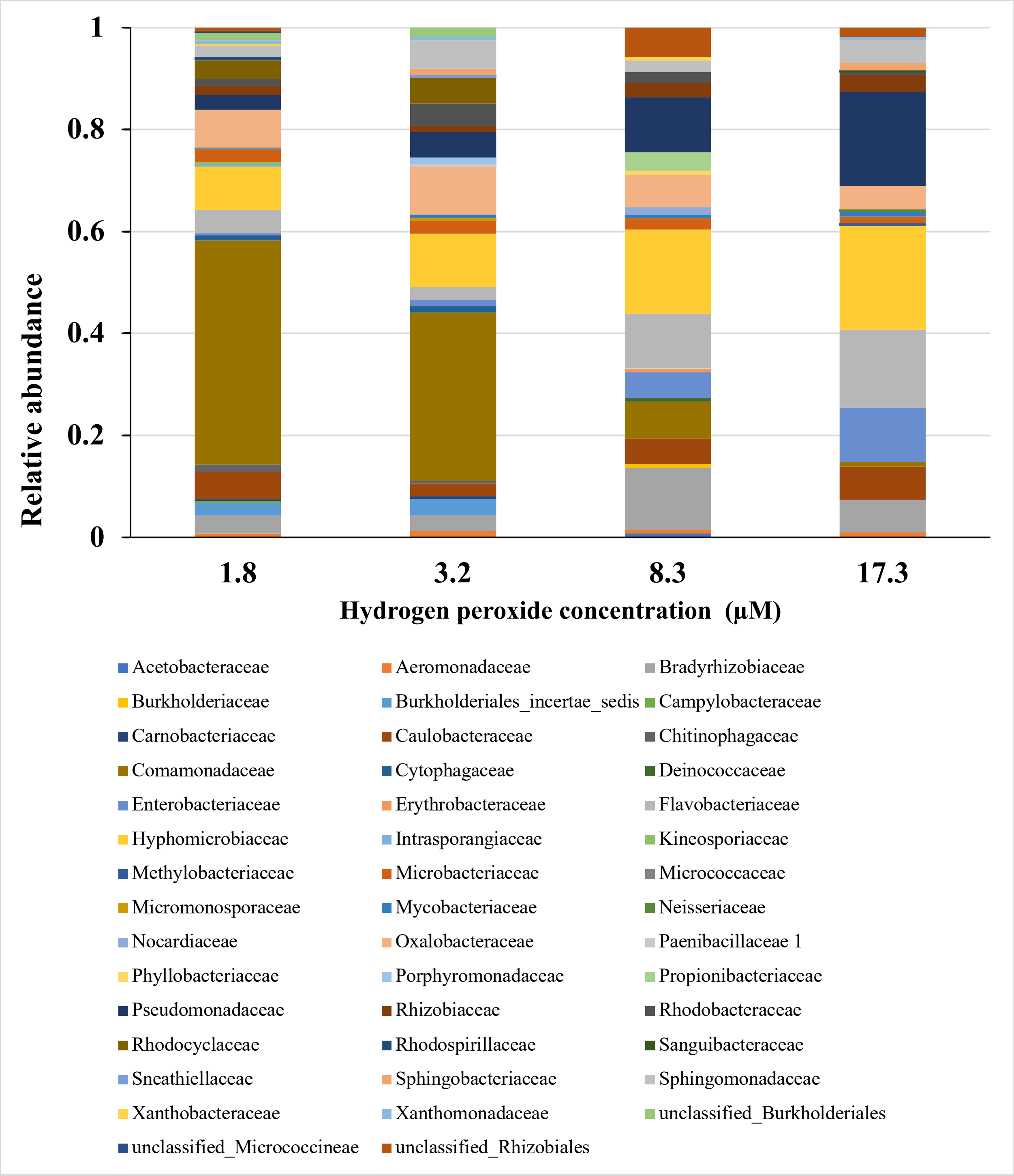

Relative abundance
0
0.2
0.4
0.6
0.8
1
1.8
3.2
8.3
17.3
Hydrogen peroxide concentration (μM)
Acetobacteraceae
Aeromonadaceae
Bradyrhizobiaceae
Burkholderiaceae
Burkholderiales_incertae_sedis
Campylobacteraceae
Carnobacteriaceae
Caulobacteraceae
Chitinophagaceae
Comamonadaceae
Cytophagaceae
Deinococcaceae
Enterobacteriaceae
Erythrobacteraceae
Flavobacteriaceae
Hyphomicrobiaceae
Intrasporangiaceae
Kineosporiaceae
Methylobacteriaceae
Microbacteriaceae
Micrococcaceae
Micromonosporaceae
Mycobacteriaceae
Neisseriaceae
Nocardiaceae
Oxalobacteraceae
Paenibacillaceae 1
Phyllobacteriaceae
Porphyromonadaceae
Propionibacteriaceae
Pseudomonadaceae
Rhizobiaceae
Rhodobacteraceae
Rhodocyclaceae
Rhodospirillaceae
Sanguibacteraceae
Sneathiellaceae
Sphingobacteriaceae
Sphingomonadaceae
Xanthobacteraceae
Xanthomonadaceae
unclassified_Burkholderiales
unclassified_Micrococcineae
unclassified_Rhizobiales

FIG S1 Phylogenetic distribution of the identified colonies classified at the family level. At least 100 colonies were randomly selected from the plates with four different H_2O_2 concentrations and subsequently identified by analyzing the amplified partial sequence of 16S rRNA gene using RDP classifier.

TABLE S1 The count of identified colonies which was used to plot Fig. S1.

**Family**	**H_2O_2 concentration (µM)**			
	**1.8**	**3.2**	**8.3**	**17.3**
Acetobacteraceae	0	0	1	0
Aeromonadaceae	2	2	1	2
Bradyrhizobiaceae	10	5	17	14
Burkholderiaceae	0	0	1	0
Burkholderiales_incertae_sedis	7	5	0	0
Campylobacteraceae	1	0	0	0
Carnobacteriaceae	1	1	0	0
Caulobacteraceae	15	4	7	14
Chitinophagaceae	4	1	0	0
Comamonadaceae	123	53	10	2
Cytophagaceae	3	2	0	0
Deinococcaceae	0	0	1	0
Enterobacteriaceae	1	2	7	23
Erythrobacteraceae	0	0	1	0
Flavobacteriaceae	13	4	15	33
Hyphomicrobiaceae	24	17	23	44
Intrasporangiaceae	1	0	0	0
Kineosporiaceae	1	0	0	0
Methylobacteriaceae	0	0	0	1
Microbacteriaceae	7	4	3	3
Micrococcaceae	1	0	0	0
Micromonosporaceae	0	1	0	0
Mycobacteriaceae	0	1	1	2
Neisseriaceae	0	0	0	1
Nocardiaceae	0	0	2	0
Oxalobacteraceae	21	15	9	10
Paenibacillaceae 1	0	1	0	0

Phyllobacteriaceae	0	0	1	0
Porphyromonadaceae	0	2	0	0
Propionibacteriaceae	0	0	5	0
Pseudomonadaceae	8	8	15	40
Rhizobiaceae	5	2	4	7
Rhodobacteraceae	4	7	3	1
Rhodocyclaceae	10	8	0	0
Rhodospirillaceae	2	0	0	0
Sanguibacteraceae	0	0	0	1
Sneathiellaceae	0	1	0	0
Sphingobacteriaceae	0	2	0	3
Sphingomonadaceae	6	9	3	10
Xanthobacteraceae	1	0	1	0
Xanthomonadaceae	2	1	0	1
unclassified_Burkholderiales	4	3	0	0
unclassified_Micrococcineae	1	0	0	0
unclassified_Rhizobiales	2	0	8	4
